# The German East-West Mortality Difference: Two Crossovers Driven by Smoking

**DOI:** 10.1007/s13524-017-0577-z

**Published:** 2017-05-10

**Authors:** Tobias Vogt, Alyson van Raalte, Pavel Grigoriev, Mikko Myrskylä

**Affiliations:** 1 Max Planck Institute for Demographic Research, Konrad-Zuse-Str.1, 18057 Rostock, Germany; 2 London School of Economics and Political Science, London, UK; 3 University of Helsinki, Helsinki, Finland

**Keywords:** Smoking, Mortality forecast, Germany, Convergence

## Abstract

Before the fall of the Berlin Wall, mortality was considerably higher in the former East Germany than in West Germany. The gap narrowed rapidly after German reunification. The convergence was particularly strong for women, to the point that Eastern women aged 50–69 now have lower mortality despite lower incomes and worse overall living conditions. Prior research has shown that lower smoking rates among East German female cohorts born in the 1940s and 1950s were a major contributor to this crossover. However, after 1990, smoking behavior changed dramatically, with higher smoking intensity observed among women in the eastern part of Germany. We forecast the impact of this changing smoking behavior on East-West mortality differences and find that the higher smoking rates among younger East German cohorts will reverse their contemporary mortality advantage. Mortality forecasting methods that do not account for smoking would, perhaps misleadingly, forecast a growing mortality advantage for East German women. Experience from other countries shows that smoking can be effectively reduced by strict anti-smoking policies. Instead, East Germany is becoming an example warning of the consequences of weakening anti-smoking policies and changing behavioral norms.

## Introduction

Germany was divided into East and West from 1949 until the fall of the Berlin Wall and subsequent reunification in 1990. Over the decades of separation, the East increasingly lagged behind the West in living standards, health care, and ultimately life expectancy. By 1989, East German life expectancy was 2.4 and 2.6 years less than that of the West for men and women, respectively (Human Mortality Database 2016). Since the reunification 25 years ago, East Germans have experienced remarkable mortality improvements. Among women, the life expectancy difference has practically disappeared, to 0.1 years in 2013; among men, the gap narrowed to 1.2 years in 2013.

The pre-reunification mortality difference was mostly due to higher incidence of cardiovascular and respiratory diseases (Höhn and Pollard 1991)—a pattern that is usually attributed to differences in the economic, social, and medical environments (Diehl 2008; Dinkel 2000; Gjonça et al. 2000; Luy 2004). However, the factors driving the post-reunification mortality convergence are less well understood. Reunification brought major changes to the living conditions of East Germans, and the relative importance of the various factors continues to be debated (Diehl 2008; Kibele 2012). Following the adoption of the West German social, economic, and political system, East Germans benefited from access to a modern Western health system that helped to reduce circulatory mortality as the prime cause of death. Likewise, East Germans witnessed a manifold increase in nominal income and purchasing power after the West German currency was introduced at a highly beneficial exchange rate of 1:1. Social expenditures on health care and pensions converged to reach the generous Western level, which helped to reduce the mortality differences between the two parts of Germany (Vogt and Kluge 2015). In addition to improved living standards and the adoption of Western health care technology, the convergence may have been driven in part by decreases in psychosocial stress resulting from the deprived living and working conditions in the East (Cockerham 1999; Häussler et al. 1995; Riphahn 1999).

**Fig. 1:**
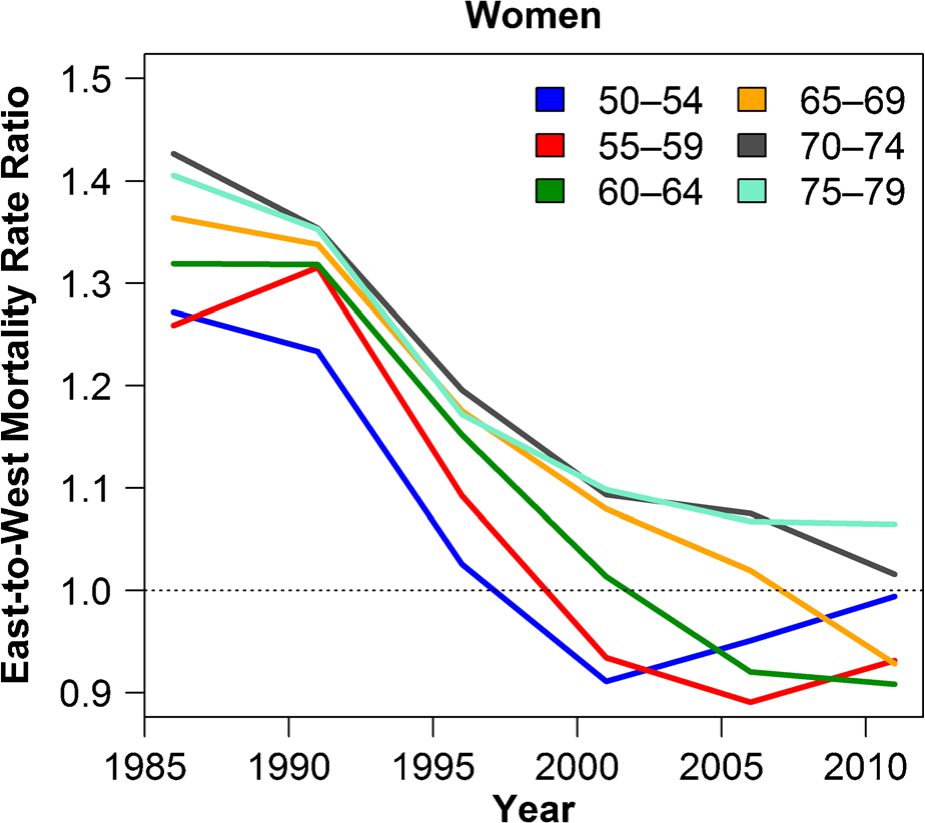
Mortality rate ratios for all-cause mortality showing convergence and crossover for women in East and West Germany. The equivalent figures for men can be found in the appendix. Ratios above 1 indicate higher mortality for East German women. Rate ratios are calculated over five-year periods from 1984–1988 to 2009–2013, centered on the middle year. *Source:* HMD (2016)

Despite these improvements, East Germany^1^ continues to lag behind West Germany in terms of living standards. However, for some age groups, female mortality has declined below that of West Germany, as shown in Fig. 1.^2^ This mortality crossover has been documented before and attributed not to period changes following the reunification but instead to higher smoking prevalence among West German women in the 1940s and 1950s birth cohorts (Myrskylä and Scholz 2013). This finding is not surprising given that smoking is the most important behavioral factor influencing mortality and is one of the key elements in contributing to mortality differences across (Preston et al. 2010) and within national populations (Preston and Wang 2006). Other health behaviors may also be partially responsible for the convergence, but prior research suggests that, at least for alcohol consumption, the trends declined in parallel in both East and West over the 1990s (Bloomfield et al. 2005).

Women in East and West Germany exhibited different smoking patterns before and after reunification, while for men the patterns remained similar. East German women smoked little during separation, when prevalence was high among East and West German men and rising among West German women. This changed abruptly after reunification. The East German smoking prevalence for women over ages 25–69 increased by 42 % between 1992 and 1998 alone, at a time when the prevalence was constant among West German women (Junge and Nagel 1999). Among younger cohorts, smoking initiation has become higher and cessation rates have become lower for East German women compared with West German women in recent years (Junge and Nagel 1999).

Traditional mortality forecasting methods that do not account for smoking would forecast a widening mortality advantage for East German women,^3^ which seems implausible given that younger East German women now smoke at higher rates than West German women and continue to lag behind in living standards. Our current forecasts incorporate smoking information. We use a two-step method in which we first project lung cancer mortality based on observed smoking histories, and then use the macro-statistical relationship between lung cancer mortality and mortality from other causes to project mortality from other causes than lung cancer (Preston et al. 2014). These results suggest a second crossover in mortality rates, with East German women forecasted to return to higher mortality than West German women for cohorts coming of age post-reunification. The results have important implications for understanding post-reunification and future East-West mortality dynamics in Germany.

## Data and Methods

### Data

We used the German Socioeconomic Panel (SOEP) (DIW Berlin 2015) to estimate cumulative years smoked by age 40 among East and West German cohorts. The question about the uptake and cessation of smoking was asked in waves 2002 and 2012, which allowed us to obtain precise information on the average number of years smoked before age 40 for each cohort born between 1922 and 1972. For cohorts born between 1973 and 1992, we predicted the cumulative smoking years at age 40 based on the cumulative number of years of being a smoker at earlier ages and the smoking experiences of older cohorts (see Wang and Preston 2009 for more detail). We smoothed the cohort smoking histories using Lowess (Cleveland 1979).

Our mortality data came from four sources. Lung cancer death counts for Western Germany came from the World Health Organization (WHO) mortality database (WHO 2014) for years 1956–1990 and from the Federal German Statistical Office via the German health monitoring website (GBE-Bund) (Federal Statistical Office 2015) for 1991–2013. We reconstructed the time series of lung cancer death counts in East Germany for the years 1956–1997.^4^ From 1998 to 2013, Eastern data was also obtained from the German health monitoring website. Further adjustments were made to the lung cancer death counts to correct for the inclusion of larynx cancer (1991–1997 in West; 1979 in East) and to separate East and West Berlin (1998–2013).^5^ Population exposure was retrieved from the Human Mortality Database (HMD) for years 1956–2013.

Lung cancer death rates were calculated for each year, 1956–2013, by five-year age groups (ages 50–54 to ages 75–79).^6^ These were the ages at which changes in smoking habits were expected to make the largest differences to future all-cause mortality. Moreover, at older ages, there is some question about the validity of estimates regarding the relationship between lung cancer and all-cause mortality for females from the Preston-Glei-Wilmoth (PGW) method that we used (Preston et al. 2010, 2011; Rostron 2010). The period trends in lung cancer were smooth in both East and West, and no breaks could be found over years transitioning between data sources or International Classification of Diseases (ICD) codes.

## Forecasting Mortality Using Cohort Smoking Histories

The method that we used is based on the Preston et al. (2014) two-step projection method. The method first projects lung cancer mortality and then bases all-cause mortality projections on the macro-statistical relationship between lung cancer mortality and mortality from other causes.

The first step—projecting lung cancer—is a modification of the Wang and Preston (2009) methodology. Rather than predicting the relationship between all-cause mortality and the cohort smoking years, *S* ^*c*^, Preston et al. (2014) suggested using the following negative binomial regression equation to estimate the relationship between lung cancer and cohort smoking patterns:

1 $$ \ln \left({M}_a^c\right)= A+{\upbeta}_a{X}_a+{\upbeta}_s \ln \left({S}^c\right), $$

where ${M}_a^c$ is the lung cancer death rate at age *a* in cohort *c*; *X*_*a*_ is an indicator of age category *a*, and β_*a*_ is its coefficient; and β_*s*_ is the coefficient of ln(*S* ^*c*^), the log of the mean cumulative number of years a cohort has smoked by the age of 40.

We created a data frame that combined the information on cohort smoking histories, with observed lung cancer arranged by age, period, and cohort into five-year categories as described in Preston et al. (2014: footnote 4). We combined East and West German data to fit the Model 1 because we wanted to increase statistical power and because we had no reason to believe that the relationship between the cumulative number of years smoked and lung cancer would differ between East and West. Our estimated β_*s*_ was 0.981. This was remarkably close to the 0.929 β_*s*_ that Preston et al. (2014) estimated for American females.

To validate our self-reported data on smoking from the German SOEP, we reestimated the Eq. (1) for East and West Germany separately, substituting *S* ^*c*^ with cohort dummy variables in an age-cohort model following Preston et al.’s (2014) second equation. The estimated cohort coefficients lined up reasonably well with the self-reported smoking histories over cohorts (Fig. 6 in the appendix).

Using the coefficients estimated from Eq. (1), we forecasted lung cancer death rates separately for East and West Germany up to the period 2031–2036, when our youngest partially observed cohorts turned 40. The method performed well over the selected age range. No discernable jump was noticed during the jump-off period (shown in upcoming Fig. 3). Cohort patterns can be discerned in the pattern of temporal change. For instance, among West German women, a first lung cancer peak can be seen in the early 1980s for 60- to 64-year-olds. This peak becomes more pronounced over each successive five-year age interval.

As a second step, Preston et al. (2014) translated lung cancer forecasts to all-cause mortality by using the PGW method (Preston et al. 2010, 2011), which estimates the macro-statistical relationship between lung cancer mortality and mortality from all other causes based on 21 countries over the period 1950–2006. The model includes effects for age, sex, period, and country as well as interactions among them. Our approach to translating the lung cancer mortality rate to mortality from other causes of death follows the Preston et al. method in spirit but departs in detail. We adopted a modified approach for three reasons.

First, the 21 countries on which the PGW method is based do not include Germany, so the validity of the translation coefficients is questionable for our purposes. We use German data to estimate comparable translation coefficients. We restrict the period from which we estimate the coefficients to the years 1991–2013 in order to avoid complications arising from the sharp changes in mortality trends before and after reunification.

Second, direct implementation of the PGW method would result in a jump in the first forecast year. We translated the forecasting problem from forecasting the log of mortality from non-lung cancer causes ln(*Mo*) to forecasting the change in the East-West difference:

$$ \ln \left(\frac{Mo_t^{East}}{Mo_t^{West}}\right) - \ln \left(\frac{Mo_{t - 1}^{East}}{Mo_{t - 1}^{West}}\right). $$

Using this approach avoids jumps in the first forecast year and reduces the complexity of the forecasting model considerably, as explained later herein.

Third, we adopted the most parsimonious defensible regression model that links lung cancer mortality to non-lung cancer mortality. We did so because the database for estimating the relationship between lung cancer and other causes of death is much smaller than in the original PGW method: only two regions (East and West Germany) and 22 years. Thus, instead of fitting categorical age and categorical age × lung cancer interactions, we used quadratic age for both. Instead of fitting annual coefficients, we fit a linear time trend, although we allowed the trend to differ between East and West.^7^ Moreover, we did not include the interaction between lung cancer and time because this was not significant. Fenelon and Preston (2012) used a PGW-type approach to analyze smoking-attributable mortality in the United States and dropped the interaction between lung cancer and time for similar reasons. The fit of our model was good, with *R* ^2^ = .998.

Figure 8 in the appendix shows the estimated coefficients that we used. For ages close to 50, these are slightly higher than those given in the PGW papers, which might be expected given that the smoking epidemic is comparatively recent among German women by western standards. Fenelon and Preston (2012) found lower coefficients when the estimation was restricted to American women, which they attributed to the maturity of the smoking epidemic there. As a robustness check, we estimate our results also using coefficients that are 50 % smaller than our preferred coefficients.

We used the PGW approach, with coefficients based on German data, to translate the forecasted lung cancer death rates into East-West differences in mortality from other causes of death. We assumed that overall mortality trends that are not influenced by smoking will be similar in the East and West in the future. This is a conservative assumption because the East has already caught up with the West on all major causes of death,^8^ and it would be bold to assume that mortality in the East will continue to decline faster than in the West. With this assumption, the one-step-ahead forecast for the East-West mortality rate difference (in %) for causes other than lung cancer is:

$$ \ln \left(\frac{Mo_{t + 1}^{East}}{Mo_{t + 1}^{West}}\right)= \ln \left(\frac{Mo_t^{East}}{Mo_t^{West}}\right)+\upbeta \left[\left({ML}_{t + 1}^{East} - {ML}_{t + 1}^{West}\right)-\left({ML}_t^{East} - {ML}_t^{West}\right)\right], $$

where ${Mo}_{t+1}^{East}$ and ${Mo}_{t+1}^{West}$ are the one-step-ahead forecasts of non-lung cancer mortality, *Mo,* for East and West; ${Mo}_t^{East}$ and ${Mo}_t^{West}$ are the same for the observed period *t*; β is the age-specific coefficient shown in Fig. 8 in the appendix; and ${ML}_{t+1}^{East}$ and ${ML}_{t+1}^{West}$ are lung cancer mortality rates for East and West.

Recursive use of the one-step-ahead forecast gives us forecasts up to the period 2031–2036. The logic in the preceding equation is to use changes in lung cancer mortality difference between East and West to inform changes in mortality from other causes between East and West. As seen from the preceding equation, the number of unknowns is small. The limitation from using this simple equation is that we do not forecast levels of mortality, only differences.

## Results

**Fig. 2:**
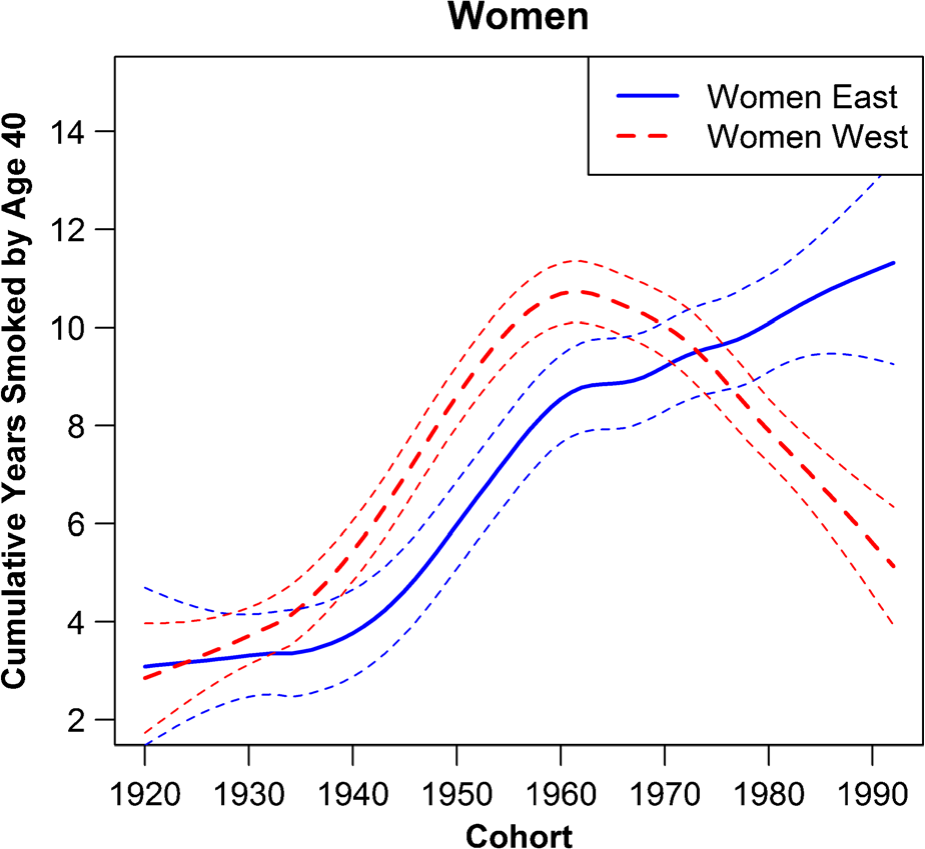
Cumulative years smoked by the age of 40 (smoothed) by birth year, with 95 % confidence intervals. The last fully observed cohort was born in 1972. We forecast the cumulative smoking years for the younger cohorts based on the smoking patterns of older cohorts and the years smoked by each cohort at least by age 20. Therefore, our final cohort, born in 1992, reached age 20 in 2012. The equivalent figure for men is in the appendix. *Source:* DIW Berlin (2015)

Figure 2 shows the observed and partially forecasted cumulative number of years smoked by age 40 for the East and West German female cohorts born between 1935 and 1992. Among all female cohorts born before the year 1950, the cumulative years smoked are considerably lower in the East than in the West. For more recent cohorts, however, the number of years smoked is higher for East than West German women. The cumulative years of smoking peaked for West Germans around the 1960 birth cohort, but continued to increase among East German women.

**Fig. 3:**
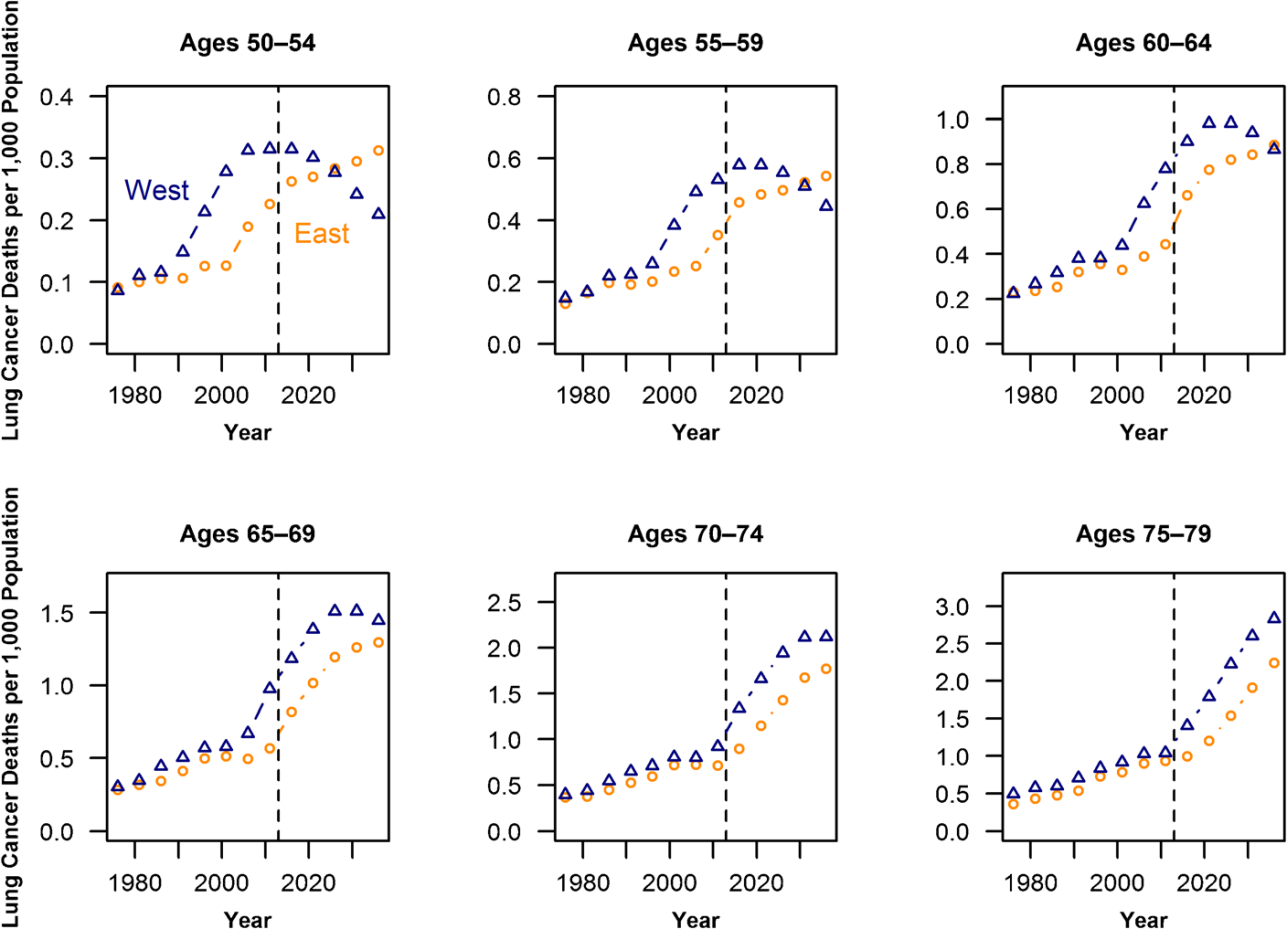
Lung cancer death rates for East and West German women (observed 1974–1978 to 2009–2013, forecasted 2014–2018 to 2034–2038) first showing divergence, followed by convergence or even crossover. Note the changing *y*-axis due to different lung cancer mortality levels by age

Figure 3 depicts observed and forecasted lung cancer mortality rates for East and West German women by age. In most age groups and years, lung cancer mortality is higher in the West in the observed data. However, for the age groups 50–64, for which earlier research has shown that total mortality is lower in the East than in the West, the lung cancer mortality difference is projected to narrow and eventually reverse; lung cancer is forecast to increase in the East as a result of increased smoking and is declining in the West. The crossover is expected to happen first for the youngest age groups, approximately by the period 2024–2028 for ages 50–54; by approximately 2034–2038, we project that East German lung cancer mortality will also catch up with that of the West for those aged 60–64. For the older age groups, we forecast continued higher lung cancer mortality in the West. Eastern women at older ages benefit from lower smoking rates in the past; thus, they have a comparatively reduced risk of dying from lung cancer in the future.

**Fig. 4.**
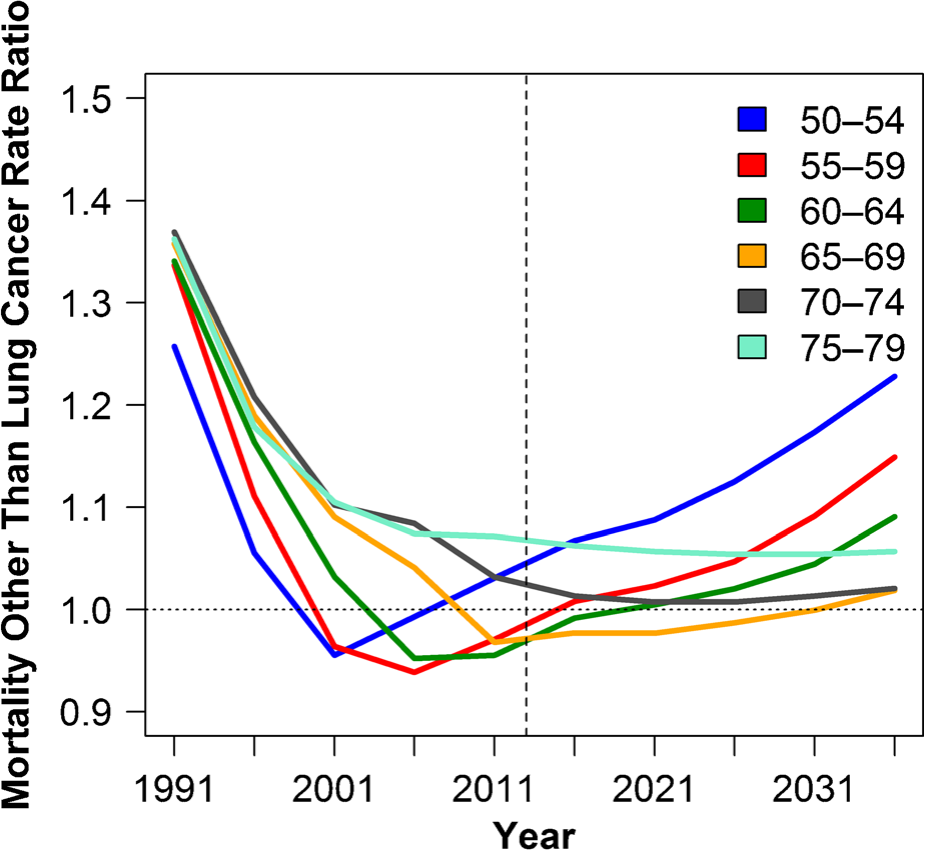
Observed and forecasted mortality rate ratios from causes other than lung cancer between East and West German women. Ratios above 1 indicate higher mortality for East Germany. Data are aggregated in five-year intervals centered on the depicted year

**Fig. 6:**
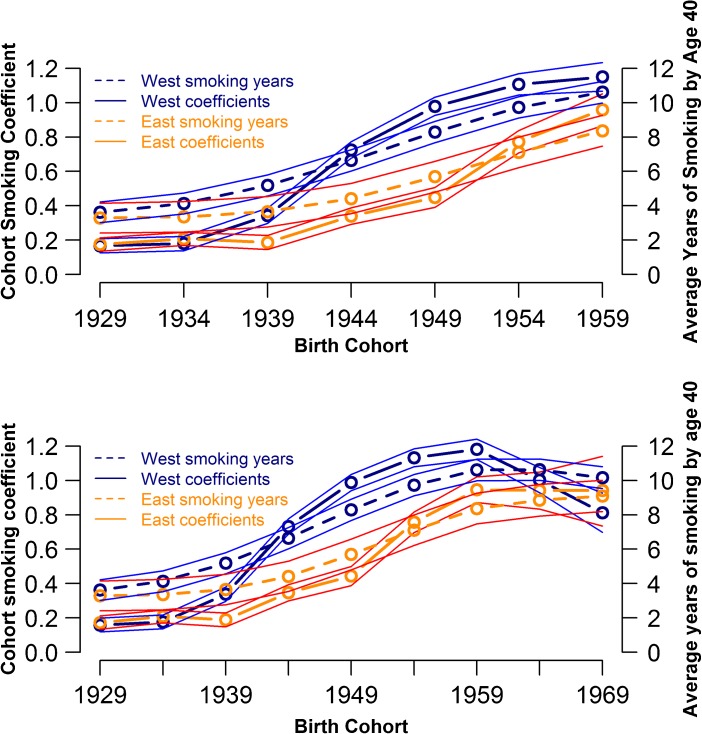
A comparison of the cohort smoking coefficients obtained using Preston et al.’s (2014:40) second regression equation (left *y*-axis) and the mean cumulative number of years that each cohort spent as a smoker by the age of 40, based on data averaged over the two SOEP rounds for cohorts fully observed to at least age 50 (above) and age 40 (below). The birth cohorts are in five-year intervals centered on the middle of each cohort. What is noteworthy is that the regression equation was based on cohort lung cancer deaths and did not include self-reported data from the SOEP. The crossover in smoking harm appears earlier for lung cancer than for SOEP data, but because these cohorts have not yet reached the age where lung cancer mortality is highest, the uncertainty in the precise timing of crossover is large. Nevertheless, the broadly similar time trends shown by the two sets of lines gives us confidence in the accuracy of our SOEP data, which we used to project lung cancer up to 2034–2038

These different lung cancer risk patterns are a key marker of the harm done by cigarette smoking to different age groups over different periods. Nevertheless, smokers have elevated risks of mortality from many other causes of death, notably circulatory disease and some cancers. We forecasted mortality differences in East and West Germany for all causes other than lung cancer, which include the bulk of smoking-attributable mortality as well as mortality not attributable to smoking, by accounting for differences in smoking histories in the two regions. Mortality from causes other than lung cancer among East German women aged 50–64 declined below that of West German women in the late 1990s and early 2000s. Our forecasts suggest that this advantage is at risk given the very different smoking patterns for cohorts born in the 1980s and 1990s (Fig. 4). Non-lung cancer mortality among East German women aged 50–54 has already surpassed that in the West; and for the age groups 55–59 and 60–64, the second crossover is projected to happen by approximately 2014–2018 and 2019–2023, respectively.

Our forecasting methodology allows us to forecast only East-West mortality rate differences for causes other than lung cancer, not actual levels. As a result, we are unable to give a precise forecast of all-cause mortality levels or the difference. However, if we combine the qualitative message of these mortality forecasts for causes other than lung cancer (Fig. 4) with those for lung cancer (Fig. 2), we can expect that for total mortality, the East will fall behind the West in the age group 50–54 by 2020 at the latest. For age groups 55–59 and 60–64, the second crossover for total mortality is expected to happen around the years 2025 and 2030, respectively, at the latest. For the age group 70–79, we expect that mortality from causes other than lung cancer, as well as all-cause mortality, will continue to be higher in the East than in the West (Fig. 4).

## Discussion

After the reunification in 1990, the German East-West mortality difference narrowed rapidly for women; by the late 1990s and early 2000s, mortality in the age group 50–64 had fallen below that of the West. This mortality crossover has been attributed to the higher smoking of the West German cohorts. In this study, we show that the smoking differential has reversed and use demographic forecasting methods to study the implications of the reversing smoking advantage to mortality differences between East and West German women. Our results show that the increases in smoking rates among younger cohorts will have a strong impact on future mortality differences. In the next two decades, East German women aged 50–69, who currently enjoy a lower mortality than their West German peers, will again fall behind West Germany in terms of both lung cancer mortality and all-cause mortality.

Past research on the post-reunification mortality differences between the East and the West have often treated the reunification as a “natural experiment” and interpreted the post-reunification changes in mortality as arising from factors related to the reunification (Chruscz 1992; Cockerham 1999; Dinkel 1992; Häussler et al. 1995; Vaupel et al. 2003). Four main factors are often considered as responsible for the sharp decline in post-reunification mortality in the East: medical care, living standards, psychosocial stress, and health behaviors (for a review, see Diehl 2008; Kibele 2012). Each of these four factors may have been important in the past convergence. However, there have been only convergences, not crossovers, on these factors. Smoking, by contrast, shows a fluctuating pattern across East and West female German cohorts. Because smoking is the most important behavioral factor influencing mortality, we would expect these changes to help explain past crossovers in mortality and also inform us about future changes. The effect of smoking on mortality is lagged, with a gap of two to three decades between a population-level increase in smoking prevalence and a marked increase in smoking-related mortality. This lag in the effect from smoking to mortality is critically important and useful for forecasting because we can use observed smoking behavior to forecast future mortality rates. Apart from smoking and the period effects related to reunification, selection effects pertaining to the years of the German separation may be responsible for the observed mortality convergence. However, a recent study by Vogt and Missov (forthcoming) found that selection played only a minor role in the post-reunification mortality convergence compared with the impact from overall improvements in the social and medical environment.

Our results suggest that the mortality advantage of the Eastern 50- to 69-year-old age group will disappear as the mortality improvement in the East, in comparison with the West, is suppressed by increasing smoking. Our prediction rests on the assumptions that the general living conditions in terms of health care, wealth, or pollution will not diverge again in the future. This is a reasonable assumption because we do not expect the two former parts of Germany to follow different political, social, or economic paths as they did in the past. There might still be regional differences in these realms 25 years after reunification, but there is a growing differentiation as a North-South pattern replaces the general East-West difference (Kibele et al. 2015).

An implicit assumption underlying indirect methods of estimating smoking-attributable mortality from lung cancer is that the lag in mortality between smoking and lung cancer death does not differ from that between smoking and other smoking-related causes of death. Available evidence, however, suggests that excess mortality among smokers occurs earlier in life from vascular diseases (such as coronary heart disease) than from lung diseases but that these excess risks also decline faster after cessation (Lopez et al. 1994). These differences in timing might cause small differences in the timing of crossover between mortality other than smoking, which includes these earlier smoking-related causes of death, and lung cancer. In our forecasts, the timing of lung cancer crossover precedes the crossover in mortality other than smoking; and using diluted coefficients (Fig. 9 in the appendix), they are well synchronized. Moreover, estimates of smoking-attributable mortality that incorporate cause-specific lag structures into a Peto-Lopez methodology differ by less than 2 % from those that do not (Oza et al. 2011). Thus, we are confident that our model has correctly identified future mortality crossovers for the 50–64 age group, although the precise period when these will happen is somewhat uncertain. A mortality crossover for ages 65–69 is less certain.

Other approaches to forecasting mortality based on smoking histories have been proposed. Wang and Preston (2009) based forecasts on the statistical relationship between all-cause mortality and smoking history. We found this approach to be overly sensitive to the inclusion or exclusion of certain cohorts. Janssen et al. (2013) suggested an age-period-cohort model that requires as input the peak smoking period by cohort. Given the immaturity of the epidemic in East Germany, there is too much uncertainty in this model.

We tested the robustness of our forecasting results. Our forecasted second crossover at ages 50–69, which is driven by differential smoking patterns, is based on a regression model that links lung cancer mortality to mortality from other causes of death. It is possible that we have overestimated the strength of this association. We conducted a robustness test that halves the original coefficients to see whether the qualitative conclusions are sensitive to changes in the model coefficients (see Fig. 9 in the appendix). Even with these diluted coefficients, we forecast that the East falls behind the West in each of the age groups between 50 and 64 in the years 2019–2033. Moreover, the age coefficients linking smoking history and mortality estimated from German data closely resemble international age coefficients estimated from 18 high-income countries in the widely used PGW model (see Fig. 8 in the appendix). We also analyzed how sensitive our results are to the assumption of complete convergence of mortality that is not related to smoking, finding that the results are reasonably robust in relation to small deviations from the full convergence assumption. The sensitivity depends on the age group. For ages 50–64, mortality that is not related to smoking would need to decline 5 % faster per decade before our finding of a second crossover by the period 2034–2038 would be compromised. For the age group 65–69, we forecast the crossover to happen around 2029–2033, and for this age group, the result is sensitive to small deviations from the assumption of convergence.

Our findings highlight the importance of policies to combat the uptake of cigarette smoking. In this respect, Germany’s reunification is an example of the severe effects of lax anti-tobacco regulations. Marketing efforts in general were not necessary in the East German planned economy because product competition and market-entry preparation did not exist (Feick and Gierl 1996). At the same time, there was no oversupply but rather shortages of certain consumer goods and services (Kopstein 2000). Rare attempts at statutory promotion gave rise to suspicion among East Germans as they believed that something must be wrong if advertisement was necessary (Feick and Gierl 1996). This pattern changed completely with the adoption of the West German free market economy, wherein marketing was a well-established feature. In the early 1990s, West Germany had very weak tobacco marketing regulations (Bornhäuser et al. 2006; Mons and Pötschke-Langer 2010), making it a “tobacco industry paradise” (Poetschke-Langer and Schunk 2001). East Germans, who were generally more credulous in relation to West German marketing (Feick and Gierl 1996), were confronted with this sudden change in tobacco marketing regulations. The availability of cigarettes and the images that advertisements or product placements convey are associated with early initiation of smoking (Biener and Siegel 2000; Dalton et al. 2003; DiFranza et al. 2006; Hanewinkel and Sargent 2008; Hanewinkel et al. 2011; Titus-Ernstoff et al. 2008).

When international tobacco companies entered the East German and Eastern European market, they used these strategies and targeted population subgroups that had previously low smoking rates (Connolly 1995; LeGresley et al. 2006). In 1991, 74 % of East German men were current or former smokers, but 64 % of women had never smoked (Robert Koch Institute 1995). Given the high smoking prevalence among men, advertising efforts that were not only unnecessary but strictly forbidden before reunification (Heinemann et al. 1995) were particularly geared toward younger age groups and women (Amos and Haglund 2000; Hafez and Ling 2005). In combination with the absence of adequate policies or preventive health campaigns, these efforts had a major impact on smoking habits in East Germany and throughout Eastern Europe.

In addition to increased exposure to cigarette marketing campaigns, reunification brought rapid and profound changes to the living environment of East Germans. A central reason why women may have reacted differently from men to the new circumstances is connected to changing gender policies and underlying gender role expectations. Before reunification, East Germany suffered notoriously from labor shortages, and women became an integral part of the labor market, working frequently in “male” industrial and agricultural jobs (Braun et al. 1994). Consequently, East German social policies helped women to reconcile work and family by providing extensive day care, financial support, and other family-friendly labor regulations (Rosenfeld et al. 2004). When the Wall fell and the East joined the West, new social and family policies brought new gender role expectations that departed from a rather conservative male breadwinner model. These changes contributed to changing marriage and fertility behaviors in the East (Adler 1997; Kreyenfeld 2003). East Germans’ age at first marriage rose steeply in the 1990s. In 1989, East Germans were 23.2 years old, on average, when they first married—2.2 years younger than West Germans. By the year 2000, East and West Germans both first married at the average age of 27.4 (Federal Institute for Population Research 2016a). This shift led to an enormous increase in the number of younger, single East Germans. For example, in 1991, 6 % of East German women and 16.5 % of West German women aged 20–34 lived alone. In 2014, 26.8 % of East German women and 21.6 % of West German women lived alone (Federal Institute for Population Research 2016b). Families and children provide social integration and control and have protective health effects (Umberson 1992). Thus, the altering demographic behavior after reunification may have contributed to changing smoking habits. Pregnancy, for example, is a time when women often quit smoking (Schneider et al. 2010). After reunification, a large number of East German women in fertile ages postponed or gave up childbearing. Between 1991 and 1996, the total fertility rate dropped below one child per women, a lower level than during both world wars (Huinink and Kreyenfeld 2006). At the same time, the mean age of mothers at first birth rose by two years from 25.2 years in 1989 to 27.2 years in 1998, compared with the small rise from 28.2 to 28.9 in West Germany (Human Fertility Database 2015). A later mean age at birth means a longer period of smoking prior to pregnancy and thus a longer time to become dependent on nicotine. A comparison of smoking habits among German women shows that in 1991, more East than West German women at all ages gave up smoking (Robert Koch Institute 2012). By 1998, this pattern had reversed, and fewer East German women gave up smoking (Robert Koch Institute 2000).

**Fig. 7:**
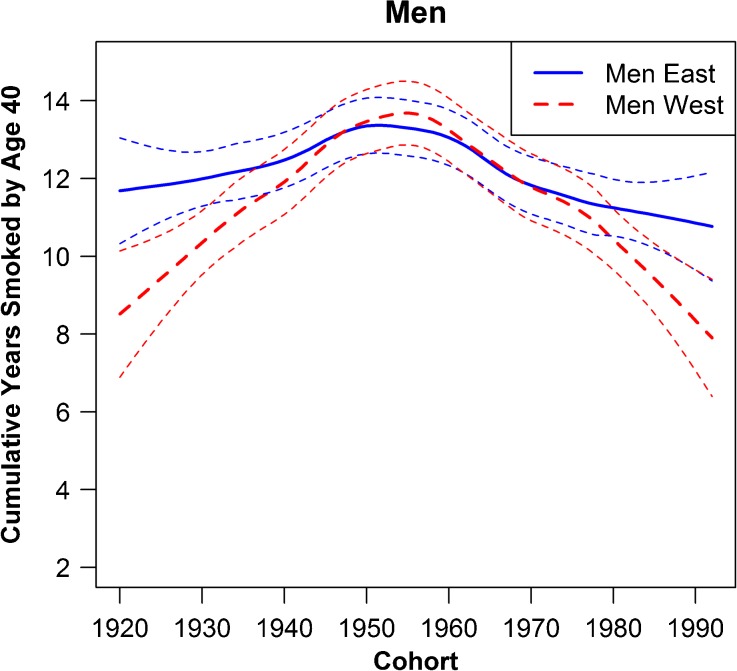
Men’s cumulative years smoked by the age of 40 (smoothed) by birth year, with 95 % confidence intervals. The last fully observed cohort was born in 1972. We forecast the cumulative smoking years for the younger cohorts based on smoking patterns of older cohorts and the years smoked by each cohort at least by age 20. Therefore, our final cohort, born in 1992, reached age 20 in 2012. *Source:* DIW Berlin (2015)

**Fig. 8:**
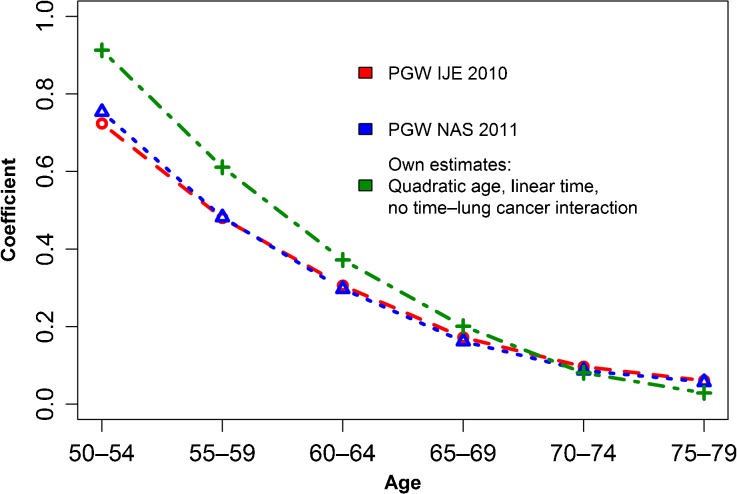
The coefficients used to estimate the relationship between lung cancer mortality and mortality from other causes of death. PGW-IJE and PGW-NAS refer to Preston et al. (2010) and Preston et al. (2011). We use the coefficients estimated using German data (own estimates)

**Fig. 9:**
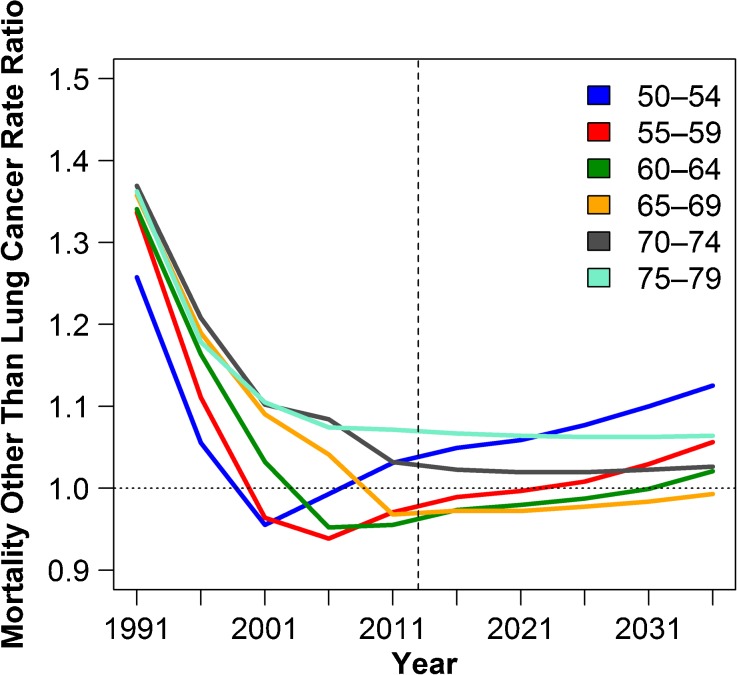
Robustness test: Observed and forecasted mortality difference from causes other than lung cancer between East and West German women. Translation of lung cancer difference to mortality from other causes based on coefficients shown in Fig. 4 multiplied by 0.5

The transformation of East German society affected fertility and marriage behavior not only through different role expectations but also very directly through bleak employment prospects and mounting economic insecurity. By 1993, 20 % of female employees were looking for new jobs (Bundesagentur für Arbeit 2015). Unemployment levels remained high throughout the 1990s and started to decline only after the 2000s. By 1994, 11% of men were unemployed; but by the year 2000, the unemployment rate for men caught up with the high level for women. Whether high unemployment had a direct impact on East German smoking levels is unclear. Weak job prospects do correlate with increased smoking initiation among female cohorts, but for younger male cohorts entering the poor labor market of the 1990s and 2000s, the smoking prevalence was lower than for older pre-reunification cohorts (Fig. 7 in the appendix). Moreover, a comparison of smoking prevalence among unemployed and employed East Germans for the year 1998 reveals that the unemployed were not more likely to smoke than the employed (Robert Koch Institute 2000), and a German panel study covering the years 1998–2008 found no direct causal effect of unemployment on smoking (Schunck and Rogge 2012). This unclear relationship may be due to conflicting reactions to a weak employment market. Recessions have been shown to both increase and decrease smoking rates. Individual-level studies have often found increases in substance use, including smoking, in reaction to economic stress and unemployment (Falba et al. 2005; Siahpush and Carlin 2006). Some population-level studies, however, have found decreases in cigarette consumption (Ásgeirsdóttir et al. 2012; Ruhm 2005). This decline in tobacco consumption might be related to decreased purchasing power brought about by unemployment or to attempts by individuals to engage in healthy behavior for fear of losing their jobs. Meanwhile, procyclical smoking behavior appears to be weakening or nonexistent for the most recently observed recessions (Nandi et al. 2013; Tekin et al. 2013). In Germany, procyclical mortality was observed over the 1980–2000 period, but behaviors were not directly examined (Neumayer 2004).

After smoking habits are established, younger age groups are more likely to start when someone in the household, their peer group, or the general neighborhood smokes (Hill et al. 2005; Kobus 2003; Lee and Cubbin 2002). The chances of becoming and staying a smoker are higher in socially deprived areas and among networks of lower socioeconomic status (Chandola et al. 2004; Gilman et al. 2003). Although life satisfaction has increased substantially in East Germany since reunification (Frijters et al. 2004), it remains lower than in the West (Easterlin and Plagnol 2008). These factors might contribute to high female smoking rates in East German regions that still suffer from continued high unemployment rates, outward migration of the more educated, and a general lack of prospects (Federal German Government 2014).

Smoking rates have long been known to vary by level of education (Escobedo and Peddicord 1996; Pampel 2005). In the German setting, socioeconomic inequalities in smoking are increasing rapidly among those born after 1941, particularly among women (Schulze and Mons 2006). German education levels vary by gender and age. The older East German female age groups that witnessed the mortality crossover during the 1990s had lower education than men but higher education than women in the West. In 1995, 52 % of East German men and 45 % of East German women in the age group 50–55 had more than 10 years of education, while comparable levels for West German men and women were 37 % and 35 %, respectively (Federal Statistical Office 2016a). As a consequence of the economic insecurity and lack of perspectives in the East, women improved their education levels more than men. These younger East German cohorts, whose cumulative smoking years are projected to surpass the number in the West, have more years of education, on average, than East German men and West German men or women (Federal Statistical Office 2016a). That these higher educational levels are not reflected in lower smoking rates is likely to reflect higher educational levels being less important than the overall macro-environment in the German setting.

Finally, we investigated whether selective East-to-West migration may have contributed to different smoking patterns in East and West Germany after reunification. For the first mortality crossover, this would be the case if leavers were more likely to smoke than those who remained, decreasing the smoking prevalence in the East while increasing it in the West; and the opposite would hold true for the second crossover. The first crossover among women aged 50 and older occurred during the 1999–2003 period. These women were above the prime age for post-reunification East-to-West migration. Thus, differential smoking behavior by migration status was unlikely to have had a large impact on the population-level East-West smoking disparities. The limited available evidence on smoking prevalence for women who are projected to have a second mortality crossover shows no relationship with migration status. According to the German Health Survey of 1998, among East-to-West female migrants of working ages 18–39, 58 % were smokers; among stayers in East Germany in this age range, 56 % were smokers, although the sample size of East-West migrants was too small to attach much certainty to the result (Robert Koch Institute 2000). Migration flows among cohorts younger than those captured by the 1998 survey (1980–1990) are still larger from East to West than from West to East but substantially smaller than in the years after reunification (Federal Statistical Office 2016b; Heiland 2004). It is not entirely clear how the intra-Germany migration pattern will change in the future for these younger women, but since the mid-1990s, migration within East Germany has overtaken migration to the West, leading to an agglomeration of cities and the increasing depopulation of rural areas (Sander 2014). Thus, any potential influence of selective outward migration of non-smokers to the West is likely to play a diminished role in the future.

Our analysis is not able to predict future all-cause mortality among German women. Yet, the aim of our study was to exemplify how plastically smoking impacts observed mortality differences. Past smoking behaviors first allowed East German women to reach a lower mortality level than women in the West. Contemporary smoking habits are projected to again reverse the difference within the next 20 years. Because smoking prevalence among women in the East increased after reunification, the low-mortality success story of East German women is anticipated to end as they return to higher overall mortality. This study gives another shocking example of how enduring the consequences of changing smoking habits are. Mainly younger East German cohorts that were exposed to the post-reunification tobacco campaigns and formerly unknown cigarette advertisements will experience higher mortality from smoking-related diseases. Thus, our findings on the role of smoking have important implications for understanding future mortality dynamics among more than 40 million German women.

## Figures and Tables

**Fig. 5:**
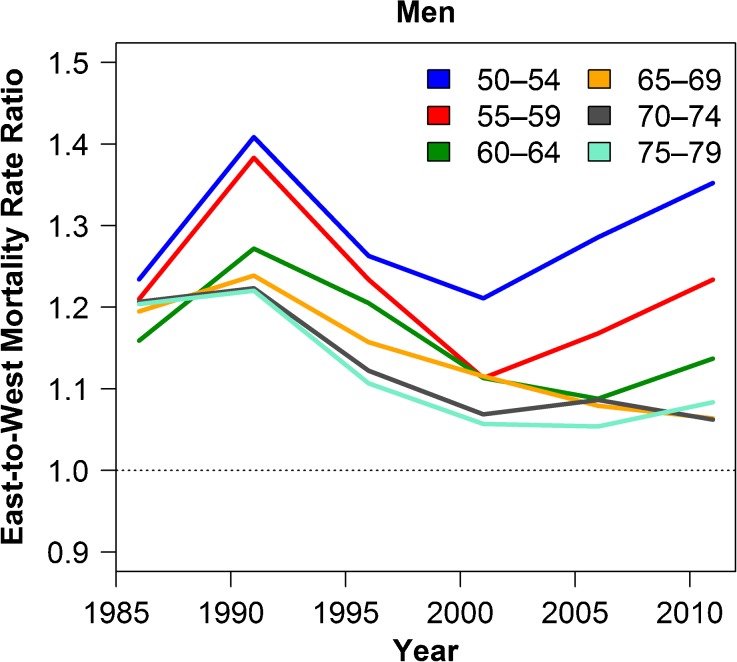
Mortality risk ratios for all-cause mortality convergence for men in East and West Germany. Ratios above 1 indicate higher mortality for East German men. Rate ratios are calculated over five-year periods from 1984–1988 to 2009–2013, centered on the middle year. *Source:* HMD (2016)

## Footnotes

1 For the purposes of the present analysis, the territory of the former German Democratic Republic (GDR) is referred to as *East Germany*. The area designated as East Germany contains the federal states of Mecklenburg-Western Pomerania, Brandenburg, Saxony-Anhalt, Saxony, Thuringia, and East Berlin.

2 This is not yet the case for men (see Fig. 5 in the appendix). As such, we focus our attention here on investigating female mortality patterns.

3 Rising differences (increasingly lower mortality in the East) were observed in Lee-Carter forecasts (Lee and Carter 1992; Lee and Miller 2001) for East and West German women aged 50–64, based on observed all-cause mortality. This was noted regardless of whether the fitting period included only the post-reunification period with high rates of mortality improvement in the East or included periods of pre-reunification stagnation. Results are not shown but are available from the authors.

4 Lung cancer was designated as the item “Malignant neoplasm of trachea, bronchus, and lung.” This item was present in all German Democratic Republic (GDR) classifications of causes of death. For the years 1956–1959, however, it did not appear as such in statistical tables. Instead, item 735 “*Bösartige Neubildung der Luftröhre, Bronchien und Lunge*” is incorporated into a broader category 73 “*Bösartige Neubildung der Ohres und des Atmungssystems*.” Following standard reconstruction procedures (Meslé and Vallin 1996), it was possible to estimate death counts for these earlier years on the basis of the composition of group 73, observed in 1960. Item 735 dominated group 73, accounting for 92 % and 79 % of all deaths for males and females, respectively. Year 1979 was special: this was the first year of the transition to a new cause-of-death classification, the ICD-9 (East). However, mortality data for 1979 were available for 35 items only. Fortunately, the item “Malignant neoplasm of larynx, trachea, bronchus, and lung,” which combines item 161 “Malignant neoplasm of larynx” and 162 “Malignant neoplasm of trachea, bronchus, and lung,” was among them. A correction factor was applied to remove larynx cancer from the 1979 data as described in the text.

5 Two adjustments needed to be made to the GBE-Bund data. First, the original data combined the ICD categories “Malignant neoplasm of larynx, trachea, bronchus and lung” for the years 1991–1997. This was also the case for the East German series in 1979. Using WHO data, we averaged the proportion of larynx cancer death counts in a combined lung and larynx cancer category by age, across the years where this was available (1973–1990 except 1979 in the East, and 1956–1990 in the West). Making the assumption that this proportion was stable over time (no discernable time trend was noticed), we applied these age-specific proportions (East and West separately) to the GBE-Bund data to subtract the larynx cancer death counts. Second, data from 1998 onward do not contain separate estimates for East and West Berlin. To split lung cancer death counts in Berlin, we assumed that the lung cancer proportions in East and West Berlin were similar to the all-cause mortality proportions in East and West Berlin. The latter were calculated by taking the difference between East and West German death counts estimated in the HMD and East and West GBE-Bund death counts, which did not include Berlin. This assumption poses greater concern for bias in the East because East Berlin makes up a larger percentage of the eastern German population than West Berlin does of western Germany.

6 Given the different data sources for the numerator and denominator, we multiplied lung cancer death counts by the ratio of all-cause mortality deaths from the numerator data source to the HMD death counts for each age and period. In most cases, the ratio was 1.0.

7 We used the West as reference. Over the period 1991–2013, the time trend coefficient for the East was –0.10 (*p* < .01), implying a faster decline in mortality that is not related to smoking. We considered alternative specifications for the time trends. In particular, we analyzed whether the East-West difference in trends should be made to interact with time, as the data suggests that the convergence is complete: if only years after 2000 are used, the difference in time trends between East and West is small and insignificant (coefficient –0.015, *p* = .43). However, our sample size is small, which calls for a parsimonious regression approach; and more nuanced approaches to modeling the East-West trend difference, such as allowing for different trends before and after year 2000, would change the key coefficients by less than 2 %. Thus, we allow the time trends to differ over the period 1991–2013; however, because most recent data suggest complete convergence, in the forecasts we assume that the trends are similar.

8 Complete or virtually complete convergence in standardized death rates between women in East and West Germany aged 50–79 was observed for cardiovascular disease, external cause mortality, and digestive diseases by the most recent year (2012). Mortality from neoplasms, infectious, and respiratory diseases is lower in East Germany. Results are available upon request.
